# The Reference Model: An Initial Use Case for COVID-19

**DOI:** 10.7759/cureus.9455

**Published:** 2020-07-29

**Authors:** Jacob Barhak

**Affiliations:** 1 Software Developer and Computational Disease Modeler, Jacob Barhak - Sole Proprietor, Austin, USA

**Keywords:** disease modeling, high performance computing, machine learning, monte-carlo, estimation, optimization, population modeling

## Abstract

The outbreak of the coronavirus disease-19 (COVID-19) pandemic has created much speculation on the behavior of the disease. Some of the questions that have been asked can be addressed by computational modeling based on the use of high-performance computing (HPC) and machine learning techniques.

The Reference Model previously used such techniques to model diabetes. The Reference Model is now used to answer a few questions on COVID-19, while changing the traditional susceptible-infected-recovered (SIR) model approach. This adaptation allows us to answer questions such as the probability of transmission per encounter, disease duration, and mortality rate. The Reference Model uses data on US infection and mortality from 52 states and territories combining multiple assumptions of human interactions to compute the best fitting parameters that explain the disease behavior for given assumptions and accumulated data from April 2020 to June 2020.

This is a preliminary report aimed at demonstrating the possible use of computational models based on computing power to aid comprehension of disease characteristics. This infrastructure can accumulate models and assumptions from multiple contributors.

## Introduction

Work reported in this manuscript attempts to start the modeling process to explain coronavirus disease-19 (COVID-19) while taking the approach of attempting to explain observed data and how well models/assumptions fit rather than predicting the future. This is preliminary work intended to demonstrate existing infrastructure to attract contributions.

The outbreak of the COVID-19 pandemic has resulted in a plethora of computational attempts to model the disease. Those attempts include: an attempt to provide a vulnerability risk score to patients according to their individual characteristics [1], attempts to model temperature effects on the virus [2], attempts to compute undocumented cases as well as duration parameters [3], attempts to predict peak active cases using two contrasting models and their combination [4], prediction of daily new cases and death in Brazil by using a statistical function [5], a well publicized prediction model that can also asses hospital resources [6], a model using machine learning techniques to fit data to multiple locations worldwide by an independent modeler with open source implementation [7], a sophisticated engine using graphical user interface and high performance computing (HPC) that can include mobility information [8], an attempt to model 100 days using a traditional susceptible-infected-recovered (SIR) approach applied to multiple countries worldwide [9], another application of the SIR model for Cape Verde Islands [10], and another differential equation based urban model that focuses on transmission through travel in different modes of transportation [11]. The US Centers for Disease Control and Prevention (CDC) has also collected and assembled an ensemble model listing many models [12]. However, despite all these publications, our knowledge of the disease is still limited. The Department of Homeland Security (DHS) compiled a summary of our current understanding of the disease [13]. This summary lists what we know and what we want to know about multiple categories related to the disease. 

Many expect that models can be used to forecast disease spread, but there is little evidence that today's models fulfill this expectation. This current outbreak has put even pressure on our confidence in their predictive power. 

If computational disease models are not predictive enough, what are they useful for? Many modelers, the author included, are trying to develop those models. Are all those model developers wasting efforts? 

For insight, it may be useful to consider other historical situations where people attempted to expand technological boundaries with little success. In the development of aviation, many early attempts were unsuccessful, yet eventually, a breakthrough occurred and then the science gradually advanced to the point where we have planes flying today as a regular phenomena. Are we in a similar situation with computational disease modeling today? 

Many computational modelers see the potential of using a machine to automate human cognitive skills. In other endeavors, humans have automated simpler tasks for activities such as playing chess, and more recently for developing driverless cars. Beating a computer in chess is already a difficult task not attainable by most humans. While the skills of a computer to drive cars are still debatable, it is already visible and many modern cars have some level of driving automation. These automated technologies have taken roughly half a century to develop as can be seen from the timelines in [14]. Comparing the timelines to the development of disease models may reveal that we are just at the start of the process of making machines that are able to comprehend healthcare. Moreover, healthcare is a much more complex problem due to the lack of data and standardization. Therefore, those initial attempts at disease modeling should not be dismissed altogether despite current failures. We should learn from these failures and design better solutions. Instead of dismissing some models, it is perhaps more prudent to look at models as assumptions being tested rather than as modern solutions that predict the future. Some of the assumptions made by models may be useful if interpreted for a machine to comprehend. If we are successful at this task, the benefits are enormous. To train one medical expert, it requires a great amount of time and effort. Once trained, their time becomes a valuable resource that is many times limited and in much demand. If we can train a machine to reason and make similar decisions by automating these cognitive skills using cheap hardware and software, the benefit to our healthcare system could be enormous both in terms of increased quality of life and in economic terms. In this context, current modelers should be viewed as explorers taking risks towards an important prize. 

However, this prize is still far since the large spread of model predictions [12] raises questions if sophisticated models are worth the effort. For example, in contrast to sophisticated models, in one very simple technique [15], eleven numbers were used to make a plausible prediction.

The large number of models listed above and by the CDC and DHS [12-13] indicate that different assumptions and models lead to different predictions. If more models are added to the list, it will be quite possible in the future to locate an outcome that a certain model predicts well and then claim that the model is good. In other words, if we have enough attempts to hit a target, we will eventually be successful. However, we cannot claim predictive power since there were many failures in the process. There is plenty of work reported. However, very little of it helps us at this point in time. Yet can we pick up the pieces and do something useful with what is left over?

Until models become more predictive, it is important to treat models as assumptions that need to be validated against observed data. The Reference Model for disease progression [16] takes this exact approach of assembling pieces of models together. It was created in 2012 to model diabetes using HPC. Machine learning techniques and an interface with ClinicalTrials.Gov were added in subsequent years. Recently it has become the most validated diabetes model known. It accumulates models and merges these with other assumptions such as human interpretations of outcomes data [17] and by using an assumption engine, it computes the combination of assumptions that best validates against observed outcomes in clinical studies. The Reference Model can now measure our cumulative computational understanding gap for diabetes. This work starts applying the same techniques to COVID-19.

## Materials and methods

The Reference Model for COVID-19 attempts to estimate multiple coefficients while trying to match model predictions to recorded outcomes for 52 states and territories. It uses a solver that optimizes those coefficients. The solver is named “assumption engine” since it deals with assumptions and models are treated as assumptions. 

Baseline population data for 52 states and territories was extracted as explained in Table 1. Sources are the US. Census 2010 [18], US. Census 2018 (table S1101) [19], Los Alamos National Lab work by Del Valle et al. [20], with supplementary information from Edmunds et al. [21], and the COVID tracking project at the Atlantic as downloaded on June 9, 2020 [22].

**Table 1:** Parameter and Sources.

Parameter	Source	Comments
Population Size	US. Census 2010	State population size from 2010 data column.
Population Density	US. Census 2010	Population density as people per square mile for each state form 2010 data column.
Family size	US. Census 2018	Mean extracted from census table S1101, for generating individuals STD was assumed to be 3 while family size must be at least 1.
Age Distribution	US. Census 2018	Generated using uniform distribution 0 to 100 optimized using evolutionary computation to match age Median per age group every 5 years and 85+ group from census table S1101.
Base Daily Interactions	Del Valle, Edmunds	Randomly generated mean was hand digitized from Figure 2 from Los Alamos National Lab work by Del Valle et al. showing interactions per age, STD extracted from Edmunds et al.
Initial Daily Interactions	Simulated	Initializes as a random uniform number between Family size and base daily Interactions to model the effect of measures taken by states by April 2020.
Days Since Start	The COVID tracking project	A parameter counting the days since first infection in a state - has no effect in this model version.
No COVID19	Simulated	Complementary to Infected and death.
COVID19 Infected	The COVID tracking project	Extracted 1st April 2020 per state. Number scaled to 10,000 virtual individuals representing the entire state while considering state population size, with at least one infected person per population.
COVID19 Recovered	Assumed	Initialized to 0 - assuming negligible number, although some recoveries were reported, it is not significant.
COVID19 Death	The COVID tracking project	Extracted 1st April 2020 per state. Number scaled to 10,000 virtual individuals per state while considering state population size.
Infection Time	Assumed	Indicating infection before simulation time. Initialized randomly if infected to a number between -15 and 0 using uniform distribution.

The baseline information is mostly gathered from US census data and from the COVID tracking project and contains an initial snapshot of the population for 52 states and territories. The population generation is optimized using object oriented and evolutionary computational techniques as described [23]. The populations are generated as 10,000 virtual individuals representing the entire state since simulations are conducted as 10,000 individuals batches, where each batch represents the entire state. All outcomes are normalized to this scale to allow efficient computation. Please note that all simulations are repeated 100 times so eventually 1,000,000 individuals are modeled per state. Results using smaller 1,000 population batches were generated and differences in results were not significant. However, 10,000 population batches allow better resolutions in results and are less prone to truncation of small infection numbers, so those were used for this paper. 

After populations are initially generated, the Monte-Carlo micro-simulation starts where each of the individuals goes through multiple phases for each time step measured as a day. Simulations are executed by the MIcro Simulation Tool (MIST) [24] that was augmented to allow infectious disease modeling and the augmented version supports the following simulation phases listed in Table 2.

**Table 2:** Simulation Phases.

Phase	Description
0 - Initialization	Executed only once at the beginning of simulation after populations are generated. Used to initialize model coefficients, initialize parameters that were not generated such as aggregate statistics, and initialize supporting parameters such as mortality probability.
1 - Pre-Transition Commands	In this simulation, this phase is empty. Generally executed each time step prior to transitions.
2 - State Transitions	Determine if state transitions occur between states according to formulas describing transition probability. This phase happens for every simulation time step.
3 - Post-Transition Commands	Adjust parameters according to current state. In this simulation, interaction levels of each individual are recalculated. Infected individuals will tend to drop to a level of interaction close to family size while non infected people will change their interaction randomly between family size and base daily interactions. Recovered individuals will go back to regular interaction level. This models the uncertainty of states closing and reopening in April-June while modeling less interactions of infected individuals. Also infection time is recorded if an individual is infected to track disease duration.
4 - Aggregate Calculations	Calculate statistics for the entire population such as the total number of interactions by all individuals and the total number of interactions by infected individuals.

**Figure 1:**
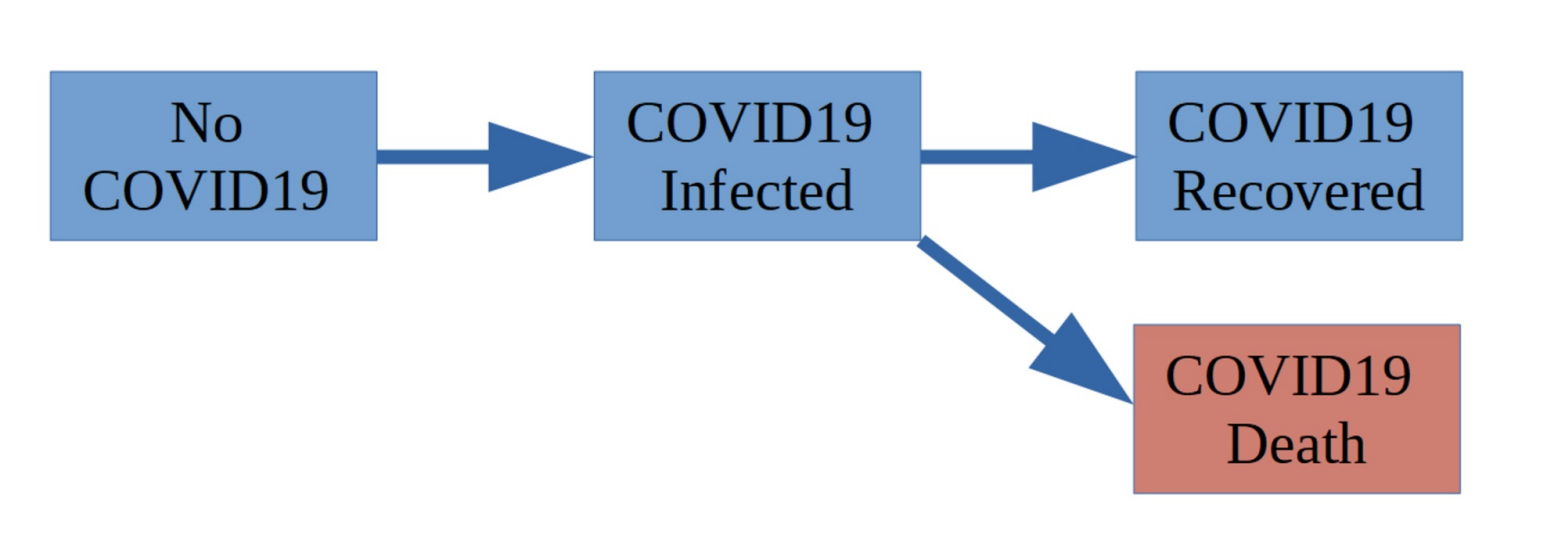
State Transition of the simple COVID-19 Model.

The main reason for simulation is to determine the state for each virtual individual at each simulation step. This happens in phase 2 according to the diagram shown in Figure 1. Arrows in the diagram represent that there is a probability to move to another state. This probability is a function that can include multiple other parameters and coefficients. Notice the important assumption that individuals who have recovered from COVID-19 cannot get infected again. The death state is colored in red and is a terminal state that stops simulation for that individual and removes the individual from the population for future time steps.

The probability to get infected in this simulation is governed by the following equation:

Min(1, (1 - (1- (Coef_Transmission*1e-2) * InfectedInteractions / TotalInteractions)** Interactions ) * (PopulationDensity / 87.4)**(0.1*Coef_PopDensity) + (Coef_OutInfect * 1e-6) )

Consider the coefficient expression Coef_Transmission*1e-2 as the probability of disease transmission from a single encounter with an infected person. This coefficient is scaled by 1/100 to keep coefficients within ranges of 0 and 1. Think about this coefficient as having percent units for simplicity. The probability of transmission by one infected person is multiplied by the prevalence of interactions of infected individuals in the population - this can easily be extracted from dividing *InfectedInteractions *by *TotalInteractions*. The combination of these products represents the probability of getting infected by one encounter. Since each individual has multiple interactions per day and any one of those can cause infection, the probability of getting infected by multiple interactions is modeled as repeated Bernoulli tests and the probability of not getting infected in one interaction is calculated by using a complementary probability. It is then raised by the power of number of interactions and again a complimentary number is applied. However, to account for differences for each state, this probability is scaled by the relative population density compared to the average and since the effect of population density is unknown, the coefficient *Coef_PopDensity *is added to control this part of the equation. The coefficient *Coef_OutInfect *is ignored in this simulation, yet in the future, it can be used to model infection outside interactions with individuals. The Min function is applied to keep the probability below 1.

The probability of recovery, in this simulation, is simplified and modeled as being infected more time than the disease duration. Formally it is coded as: *Gr(Time-InfectionTime,COVID19Duration)*.

Note that *InfectionTime *is set in Phase 3 and Time represents the number of days. The duration coefficient influences *COVID19Duration* that is calculated at Phase 0 by using the formula: 5 + Coef_Duration*30 + 3*CappedGaussian3 where *CappedGaussian3 *is a normal distribution random number capped at 3 std, and *Coef_Duration *is a coefficient initialized at Phase 0. For *Coef_Duration = 0.5* we get 20 days duration on average. However, this number will change during optimization.

The probability of death is derived from published CDC mortality tables [25] and is age dependent. In Phase 0 of the simulation, the probability of death is calculated as:

(1-(1- ((Coef_Mortality)*MortatliyTableLow+ (1-Coef_Mortality)*MortatliyTableHigh))**(1/COVID19Duration))

Where *MortatliyTableLow *and *MortatliyTableHigh *correspond to the low mortality and high mortality bounds per age group as shown in [25]. Those mortality tables are discrete functions that depend on the age of the individual. Note that a combination of two different mortality models is used here. The model combination is made by introducing the *Coef_Mortality*. If *Coef_Mortality=1* the lower bounds are used and if *Coef_Mortality=0* the higher mortality bounds are used. Every other value linearly interpolates the bounding values. The blend here is simple, only between two models, yet this blend can be easily extended in the future to multiple models arriving from different sources as done in [17]. Since those mortality numbers are for the entire duration of the disease we have to reduce those to daily probability by assuming a Bernoulli test for *COVID19Duration *days, in which the individual does not die, and take the complementary probability. We also reduce this probability to zero by multiplying with *Le(Time-InfectionTime,COVID19Duration)* to avoid conflict with the recovery probability.

Note that the model is very simplified and considers the disease duration as a preset risky period where the patient is infectious. The model does not distinguish between the latency period, incubation period, the period of communicability, and at risk of death period. There wasn’t sufficient data to feed the model so as to cross reference those assumptions in a reasonable way. The decision to keep duration simple was so it would be possible to generate preliminary results to demonstrate overall capabilities quickly. The simplicity or complexity of the model will eventually be determined by the ability of ingesting data and assumptions into the model. Future versions may address those issues as well as many other issues, potentially combining duration numbers extracted from other models such as those calculated in [3].

The reader may notice a few coefficients being used in the formulas. Those coefficients driving the simulation are being calculated by the system. In fact, the main purpose of the model is to calculate those parameters since those explain the behavior of the disease. The coefficients are listed and explained in Table 3.

**Table 3:** Model Coefficients.

Coefficient	Type	Explanation
Coef_Duration	Optimized	Coefficient controlling duration of disease
Coef_Transmission	Optimized	Coefficient related to transmission between people 0 means no infection 1 means max infection probability from meeting a person
Coef_PopDensity	Optimized	Coefficient to regulate the effect of population density
Coef_Mortality	Optimized	A coefficient interpolating the death rate between two bounds
Coef_FreeGeneral	Static	Represents the general freedom level - totally free people are not taking social protection measures
Coef_FreeInfected	Static	Represents the infected freedom level - totally free infected are not taking social protection measures and may not be aware of their situation
Coef_OutInfect	Static	A constant infection rate from infection outside the group or from causes other than interaction
Interpretation1	Special	A parameter related to human interpretation of outcomes handled separately by the system when human interpretation of outcomes is available. In this simulation, this coefficient is unused.

Static coefficients are considered as constants during this simulation while optimized coefficients are the reason for running the simulation and those will change. However, static coefficients have a potential of changing as different initial values in other simulations. This is similar to starting competitions between different model variations from different initial conditions and optimizing those in parallel, while each set or parameters competes. This can be helpful in getting closer to computing a global minimum. Yet again, in this simple simulation, static coefficients are constants. Moreover, this simulation ignores the special *Interpretation1 *parameter that deals with human interpretation of outcomes, see [17] for details.

The Reference Model compares simulation results to observed results in the 52 states and territories and will attempt to improve the fitness of results by changing model coefficients using the assumption engine. In this work, the fitness function is defined by the weighted average of the norm of the vector of differences* [infections, recoveries, deathsx100]* taken at 2 points in time - after 30 days and after 60 days. The death scaling is crucial since mortality is an important measure that is small compared to infections. So the mortality multiplication is essential to allow fitness to have a balanced influence from death and infections. Without the multiplication, coefficients tend to favor one outcome and result in a very long disease duration with low transition rates matching only infections and ignoring mortality. The reader should note that different fitness functions can produce different best models. The weights in the weighted average are according to state size. This way larger populations have more influence on results. Note that since only 10,000 people are generated per state 100 times, the infections, recoveries, and deaths compared are scaled. Thus, results are relative and the weights scale back the numbers to properly represent sizes.

The optimization method used by the assumption engine is a variant of gradient descent, a technique used for machine learning when optimizing neural networks. This technique attempts to gradually improve the fitness of the model with each iteration [17]. Since the model has stochastic elements due to the Monte-Carlo simulation and the population generation, Monte-Carlo noise exists. This noise is reduced by repeating the simulations a large number of times. However, this error will never be eliminated. Yet, it is reduced by an order of magnitude when repeating the simulations of 10,000 individuals 100 times and selecting the mean. When using the gradient descent technique, the variation around a certain solution is visible since sensitivity analysis is included as part of the algorithm when deciding on the next iteration coefficient values. This sensitivity analysis is visible in the results presented hereafter.

## Results

The results presented here were obtained by a simulation that took over nine hours on the Rescale cloud using 20 HC Series nodes with 44 cores in each node, accumulated to the total of 880 cores. This amount of computing power is necessary to calculate all variations of 10,000 individuals for over 60 time steps repeated 100 times per 52 states for multiple iterations. The many computers actually simulate many millions of individuals with multiple model variations through 5 iterations.

**Figure 2:**
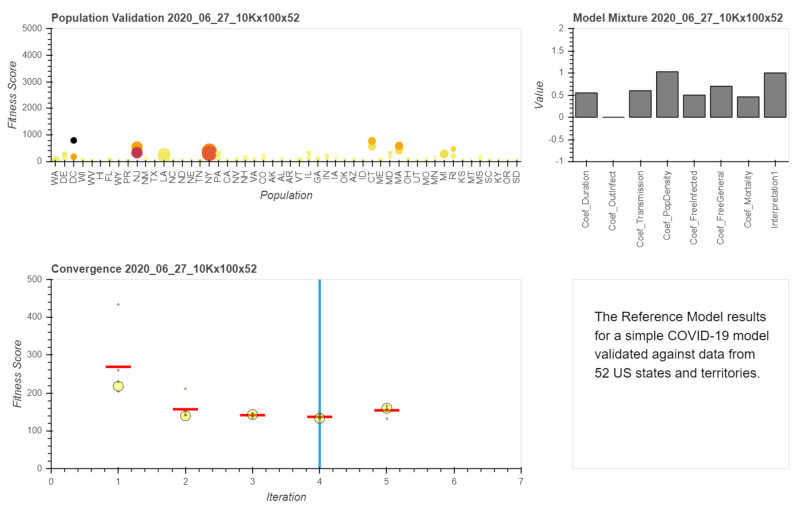
Results Summary Snapshot at Iteration 4. Upper Left - population validation plot, Upper Right - coefficient values, Bottom - convergence plot.

The large amount of information reported by the simulations is displayed in Figure 2 as a snapshot of the best fitting model in iteration 4. However, to best explore the results the reader is invited to explore the interactive version of the results [26] which also contains more details about the model. The reader should choose the Combined link [26] in using a modern web browser. Using the interactive version, the viewer can change the iteration number by sliding the slider, or change the meaning of the size and color of the circles in the population plot. In the interactive version, the reader can also hover over the graphics with a mouse to see additional information on each of the graphical representations.

The bottom part of Figure 2 shows the convergence plot where the overall weighted fitness score is presented for each iteration. The large circle represents the fitness of the unperturbed variation while the smaller circles represent the variations of the gradient descent algorithm. Those variations also serve as sensitivity analysis of the results, and when the smaller yellow circles are close to the large yellow circle this means that the solution is somewhat stable. Since these solutions are prone to Monte-Carlo error, a close batch of circles also means that the Monte-Carlo error is probably negligible. The red horizontal line represents the average of all the variations in that iteration. It can be seen that fitness and variation improve until we reach iteration 4 and then iteration 5 produces results that do not improve fitness and variations diverge. This is interpreted as an improvement in model parameters up until variation 4. Iteration 5 then diverges and this can be explained as a gradient descent step size being too large and the solution is, therefore, getting away from the local minima. Since iterations 2 to 4 show improvement and their fitness are close and iterations 3 and 4 spread are close, it is reasonable to assume that iteration 4 is stable and it will be used as the selected solution, hence the blue vertical line indicating the iteration displayed. Please note that these observations were made after many other model simulations using lower resolution with 100x1,000 individuals per state or territory. This phenomenon where there are oscillations after reaching minimum is interpreted as being near optimal. At this point, more computations are a matter of availability of computing resources and may not improve the solution much further beyond what was reached in iteration 4.

The upper left plot in Figure 2 shows the level of validations of the model to 52 US states and territories. Each column represents the state by its postal abbreviation and contains two circles representing the fitness score for that state for 30 days and 60 days. Hovering with the mouse over each state will reveal additional information such as the number of infections/deaths/recoveries compared to the observed number and state statistics such as average age. Age is used as a factor in mortality and therefore the user should be able to observe it. In fact in the current view, the size of the circle represents age while the circle color represents infections. The user viewing the interactive version can change this visualization to see non infection and death as size and color. It is clear that several states like NY are outliers for the best fitting model. However, even states close to zero may also have discrepancies and may just represent states where infection/death numbers are low and therefore the error is not high. Recall that the best fitting model works the same for all states and the only differences are in initial population statistics including age, population density, and infection levels, so the best fitting model underestimates some states and over estimates others, while attempting to find parameters that produce the best balance using the assumptions currently made in the model. To better fit the states data, more assumptions are needed to introduce more flexibility in the model function, to better represent the differences in state behavior. Clearly, the current model is not perfect, yet it is the best possible model based on assumptions made and computing resources available.

The upper right figure represents the model parameters used in the model iteration corresponding to the coefficients listed in Table 3. When exploring the interactive version of the plot, it is possible to see the change in the parameters in other iterations to see the path the gradient descent algorithm took to reach the best model. The most dominant parameter is the transmission rate that is a major driver of the simulation - it controls the individual infection rate. The transmission coefficient reached the value of 0.6 - which translates to a probability of 0.6% to contract the disease per encounter with an infected person. Once infected the average duration of the disease would be governed by the duration coefficient with the value 0.55 which will indicate that the individual would be 5 + 0.55*30 = 21.5 days from infection to recovery. This parameter fluctuates during simulation around the value of 0.5 suggesting that there may be other solutions that will fit well with a different duration coefficient. Another important coefficient is the mortality coefficient as it drives mortality in the model according to two models derived from the CDC numbers. In iteration 4 that value was 0.461 indicating slight preference of CDC higher mortality. For example for age group >85 where, CDC numbers were 10.4%-27.3%, the mortality rate would be: 0.461* 10.4 + (1- 0.461)* 27.3 ~ 19.5%. This coefficient does not change much indicating that its influence is less important than the transmission rate. The last parameter that is allowed to change is population density and it indicates the level of magnification of transmission in denser locations. It does not change much and in the future, it may be removed since the minor change may indicate that the assumption that population density has a strong impact on transmission may not be correct.

## Discussion

Although many model versions have been attempted before, the results presented here should be approached with caution since the model is very simple and does not include elements such as incubation period, infection period, and at risk of death period. Also, the numbers reported reflect the fact that from April to June governments imposed protection measures that reduced this transmission rate. Moreover, there is much uncertainty regarding the number of interactions per person. Those interactions should differ per state since different states have had different strategies to cope with the pandemic. To improve estimation, a much more accurate count of interactions is needed. In the future, it may be possible to improve interaction estimates by incorporating information on mobility [27] or from similar data sources that can help assess interactions between humans.

After incorporating more accurate data, the transmission per encounter rate will probably rise from 0.6%. However, even if the infection period is much shorter than the model assumption and the associated number calculated, this number would probably not rise dramatically if protection measures imposed by states are not lifted. This number incorporates those protection measures within it.

Despite the strong impact COVID-19 has had on society, it still has not reached the large numbers of yearly mortality imposed by heart disease. So properly calculating the per encounter infection probability may have a positive social impact by allowing administrators to assess the risk of further opening the economy.

The mortality number may be more believable since it was extracted from two possible outcomes from one source [25] and the model calculation is not very far from numbers reported by another source [28]. However, adding more mortality models to the ensemble model may make this number more accurate and may show variation of multiple age groups.

The reader should also be aware that the results reported here, although showing convergence, may indicate a local minimum and there may be many more stable solutions if the simulation starts from other initial values. Although simulation versions starting from the same initial conditions in different population scales have so far had similar behavior, simulations that start from different initial conditions may converge to a different local solution. The shape of the function optimized by the assumption engine is not known as it is constructed from many pieces of information bundled together during simulations. There may be many other possible solutions and more computing power that will allow a better exploration of this function and for extracting solutions that fit reality. The simplest thing is to try and execute the same simulation from different initial values and see if there is an agreement between converged outcomes. The Reference Model is capable of such parallel initial value exploration. However, this requires more computing power.

Looking at the large spread of errors in different states brings the question if the numbers reported are correct. The COVID tracking project provides a data quality rate for each state and provides textual information explaining the data. Hence, it is obvious that data quality can vary. There are debates on the accuracy of infection and death classification numbers with examples that suggest that the observed outcomes used by the model for validation may be incorrect, and may be under-reported [3,29] or over-reported [30].

The Reference Model is equipped with the ability to include expert interpretation of observed numbers. The *Interpretation1 *coefficient in this simulation corresponds to an expert that believes all outcome results as reported by The COVID Tracking Project - see the last bar in the upper right plot in Figure 2. This coefficient is kept constant in this preliminary version of the model since the interpretation capability was not used. If multiple experts can provide their interpretation on accuracy of the numbers, it will be possible to include those interpretations as assumptions in the optimization made by the model as explained in detail in [16]. This handling of interpretations by a machine may have advantages to the human-centric Delphi method.

The Reference Model in this work was diminished greatly and the model presented captures a very small set of its existing capabilities since this work represents an initial use case for COVID-19. The ensemble model part was minimized to only one coefficient interpolating two mortality models from the same source in the mortality transition in one disease process. This is in contrast to the diabetes version of the ensemble model 30 different models/assumptions are used for 5 transitions with multiple disease processes. However, the diabetes model took over half a decade to construct while the work on the COVID-19 model is about a quarter of a year old. Future versions of the COVID-19 model will include elements such as incorporating human interpretation, merging multiple models/assumptions regarding infection rate, additional mortality models. With more information from different sources accumulated in one location that is validated and optimized against observed outcomes will allow us to calculate how well we cumulatively computationally comprehend COVID-19.

Limitations

The reader should also be aware of limitations related to the use of synthetic data in modeling. Synthetic data may not represent unknown relations that are found in real data. However, unless those unknown relations can be modeled using assumptions, in many cases using synthetic data may be the best approach available. This work falls in this category.

Other limitations may arise from the simple model assumptions. For example, if future studies reveal reinfection of recovered patients is possible, this transition probability should be introduced in the model.

Another limitation is data availability. State data was used in this paper since this data was readily available. If higher resolution population and good quality outcomes data are available, switching to that level is a relatively easy task. The Reference Model used clinical trial populations in the past and the switch to state population data was relatively easy. So data availability is the limit rather than modeling technology.

One important current limitation that can be easily overcome today is the availability of computing power. The results reported here used modest resources. With more computing power, better results can be achieved.

Present and future

The main reason for publishing this model at its current state is to attract experts to provide feedback and potentially influence future versions of the model. Specifically, experts that can provide opinions on the accuracy of each state and experts that can provide other modeling assumptions which can be added to the model are invited. The Reference Model is an ensemble model that accumulates information and models/assumptions and finds the best fit among those. From this perspective, it is different from other models. If enough knowledge is accumulated in the system, the model fitness should gradually improve and potentially surpass human capability. 

There is the question of how human reasoning compares to machine reasoning. Being able to measure our cumulative understanding may be an important accomplishment since if we improve it every year, we will eventually reach a point where machines are comparable to humans with respect to medical reasoning. The Reference Model gives us this measure of how well our combined knowledge fits reality. The Reference Model approach and similar techniques can better guide our development.

## Conclusions

The Reference Model technology was successfully applied to COVID-19 with a very simple preliminary model. The importance of this work is less in the numbers predicted, since those numbers will change in future versions. The importance is the ability to combine multiple reported information elements in one model that can validate their combination against each other and calculate how well the information fits. This allows measuring our cumulative knowledge gap which is an important reference decision makers should have.

The Reference Model is designed as a knowledge accumulator, which is an important advantage over other models. It allows plugging in different models and assumptions as computational building blocks. In this approach, a centralized hub receives components from different sources, and computing power is used to perform the best model assembly. This paper notifies the readers that such an assembly infrastructure is now available for COVID-19.

## Appendices

Why Publish these Results Now?

There were 20 other versions before the model was executed to obtain these results. Model versions included bug fixes, engine upgrades, parameter changes, use of new data sources, different fitness definitions, different variations of initial conditions of coefficients, different date spans modeled, computational load test, and other changes. Altogether, there were over 50 simulations using the models using different population sizes and iteration lengths, many of which took considerable amounts of computing power before results were satisfactory. This version was selected since multiple simulations were stable and reported similar behavior through several simulations while computation resources were running low indicating that this is the best that can be done within time and resources allocated. Future versions will follow.

Disclaimer

The model has been developed over several months and tuned to reach the level presented in this manuscript. The model was developed to the best ability of the author. The author's experience is that there is always room for improving the latest version of an existing model and modeling errors are not rare despite precautions. The author is happy to communicate with others about the model and invites further scrutiny with the intention of publishing new results when the model version improves. This manuscript should, therefore, be considered a work in progress and it is aimed at informing the readers and possibly creating collaborations that will improve this work.

Reproducibility information

Results presented in this work are archived in the file MIST_Ref_COVID19_Cloud_2020_06_27_Plus.zip for reproducibility purposes. MIST version 0.99.4.0 was used for executing the simulation on the Rescale cloud using 20 HC Series compute nodes. An edited model printout is available in [26]. Visualization processing is archived in: ExplorationCOVID19_2020_07_02_Upload.zip.
